# Testing the Broad Definition of Pre‐Eclampsia: Should Chronic Hypertension With Fetal Growth Restriction Be Included?

**DOI:** 10.1111/1471-0528.70233

**Published:** 2026-03-25

**Authors:** Peter von Dadelszen, Terry Lee, Joel Singer, Laura A. Magee, For the CHIPS Study Group

**Affiliations:** ^1^ Department of Women and Children's Health, School of Life Course and Population Sciences King's College London London UK; ^2^ School of Population and Public Health University of British Columbia Vancouver British Columbia Canada

Historically, pre‐eclampsia was defined as proteinuric gestational hypertension or that superimposed on chronic hypertension. The 2021 International Society for the Study of Hypertension in Pregnancy (ISSHP) guidelines updated the broad definition of pre‐eclampsia [1]. One controversy has been whether women with chronic hypertension whose pregnancies are complicated by fetal growth restriction (FGR) fulfil a clinically relevant definition of pre‐eclampsia or whether FGR is solely a complication of chronic hypertension, based on the shared pathophysiology of placental dysfunction. Therefore, we examined Control of Hypertension in Pregnancy Study (CHIPS) trial data [2] to determine whether or not including pregnant women living with chronic hypertension whose pregnancies are complicated by small‐for‐gestational age (SGA) infants is at increased risk of adverse perinatal and, in particular, maternal events, compared with women with chronic hypertension who give birth to babies with birthweights at or above the 10th percentile for gestational age.

This was a secondary analysis limited to those women with chronic hypertension who participated in the international CHIPS trial in which randomisation was stratified by type of hypertension [2]. Women were included if they had non‐severe, non‐proteinuric pre‐existing/chronic hypertension; a diastolic blood pressure (dBP) of 90–105 mmHg if they were not receiving antihypertensive therapy, or 85–105 mmHg if they were receiving such treatment; and a live singleton fetus at 14^+0^ weeks to 33^+6^ weeks of gestation (determined in most cases by early pregnancy ultrasound examination). Chronic hypertension was defined as dBP ≥ 90 mmHg before pregnancy or at < 20^+0^ weeks' gestation. Women were randomised to ‘tight’ (target dBP = 85 mmHg) versus ‘less tight’ (target dBP = 100 mmHg) control. To be conservative, infants who were SGA, defined as birthweight < 10th percentile for sex and gestational age [3], were selected as a surrogate for FGR, recognising that ≈50% of SGA infants are growth‐restricted due to placental dysfunction [4]. We compared the rates of the CHIPS trial outcomes between women who gave birth to infants who were SGA, compared with those with birthweights ≥ 10th percentile, using logistic regression, with adjustment for maternal age, parity, group allocation in CHIPS and CHIPS adjustment factors (see Table footnote). A *p* < 0.05 was considered statistical significance (SAS 9.4, SAS Institute Inc., Cary, NC). No adjustment was made for multiple comparisons.

987 women participated in the CHIPS trial, of whom 727 women had chronic hypertension and known birthweights and gestational age at birth; 122/727 (16.8%) gave birth to SGA infants (Table 1). Recruited women were generally in their 30s, parous and recruited during the second trimester. About two‐thirds were on antihypertensive at randomisation, with 15% having had prior severe hypertension in the index pregnancy. 5%–6% had gestational diabetes. Women who gave birth to SGA infants were slightly older, less frequently overweight or obese and more frequently randomised to ‘tight’ control (Table 1). Women with chronic hypertension complicated by SGA (vs those without SGA) experienced significantly more often: episodes of severe hypertension; maternal manifestations of pre‐eclampsia, including new proteinuria (with all definitions provided in the Table footnotes); and preterm birth (< 34^+0^ or < 37^+0^ weeks), receipt of high‐level neonatal care ≥ 48 h, or pregnancy loss or receipt of high‐level neonatal care ≥ 48 h.

**TABLE 1:** Characteristics and effect of small‐for‐gestational birthweight on outcomes in pregnant women with chronic hypertension (*n* (%) or mean (standard deviation), with denominators shown when not as indicated at the top of the column).

	BW < 10th percentile (*n* = 122)	BW ≥ 10th percentile (*n* = 605)	OR [95% CI]	*p*	aOR [95% CI]^j^	*p*
*Baseline characteristics*						
Maternal age, mean (SD)	34.8 (5.5)	33.7 (5.6)	1.04 (1.00, 1.08]	0.041	—	—
Nulliparity	45 (36.9)	176 (29.1)	1.42 [0.95, 2.14]	0.089	—	—
Body mass index ≥ 25 kg/m^2^	83 (68.0)	486/601 (80.9)	0.50 [0.33, 0.78]	0.002	—	—
Use of antihypertensive therapy at randomisation	81 (66.4)	397 (65.6)	1.03 [0.69, 1.56]	0.870	—	—
Previous systolic BP ≥ 160 or diastolic BP ≥ 110 mmHg in this pregnancy	18 (14.8)	93 (15.4)	0.95 [0.55, 1.65]	0.863	—	—
Gestational diabetes prior to randomisation	6 (4.9)	35 (5.8)	0.84 [0.35, 2.05]	0.705	—	—
Gestational age at recruitment, mean (SD)	21.7 (6.0)	22.2 (6.0)	0.99 [0.96, 1.02]	0.456	—	—
Randomised to ‘tight’ control^a^	71 (58.2)	290 (47.9)	1.51 [1.02, 2.24]	0.039	—	—
*Pregnancy outcomes*						
Maternal						
Severe hypertension^b^	60 (49.2)	192 (31.7)	2.08 [1.40, 3.09]	< 0.001	2.52 [1.60, 3.96]	< 0.001
Any maternal manifestation of pre‐eclampsia	68/121 (56.2)	262 (43.3)	1.68 [1.13, 2.49]	0.010	1.84 [1.21, 2.81]	0.004
New proteinuria^c^	42 (34.4)	151 (25.0)	1.58 [1.04, 2.39]	0.032	1.70 [1.09, 2.66]	0.019
≥ 1 symptom of pre‐eclampsia^d^	41 (33.6)	188 (31.1)	1.12 [0.74, 1.70]	0.583	1.18 [0.76, 1.86]	0.459
Signs of pre‐eclampsia^e^	0 (0.0)	7 (1.2)	0.33 [0.02, 5.80]	0.445	—	—
Abnormal laboratory features of pre‐eclampsia^f^	7/121 (5.8)	34 (5.6)	1.09 [0.48, 2.46]	0.845	—	—
Serious maternal complications^g^	1 (0.8)	17 (2.8)	0.42 [0.08, 2.24]	0.307	—	—
Perinatal						
Gestational age at birth, mean (SD)	35.6 (4.1)	37.4 (3.0)	—	—	—	—
Delivery < 34^+0^ weeks	27 (22.1)	66 (10.9)	2.32 [1.41, 3.82]	< 0.001	2.26 [1.34, 3.83]	0.002
Delivery < 37^+0^ weeks	62 (50.8)	154 (25.5)	3.03 [2.03, 4.51]	< 0.001	3.23 [2.08, 5.02]	< 0.001
Pregnancy loss^h^	12 (9.8)	7 (1.2)	9.03 [3.56, 22.87]	< 0.001	—	—
Stillbirth	10 (8.2)	5 (0.8)	10.19 [3.55, 29.21]	< 0.001	—	—
High‐level neonatal care for > 48 h (among liveborns)^i^	(*N* = 112 liveborns) 63/112 (50.8)	(*N* = 600 liveborns) 134/600 (22.3)	4.47 [2.94, 6.80]	< 0.001	4.87 [3.04, 7.79]	< 0.001
Pregnancy loss or high‐level neonatal care for > 48 h	73 (59.8)	140 (23.1)	4.95 [3.29, 7.44]	< 0.001	5.62 [3.54, 8.91]	< 0.001

Abbreviations: aOR, adjusted odds ratio; BP, blood pressure; BW, birthweight; CI, confidence interval; OR, odds ratio.

^a^ ‘Tight’ control target diastolic blood pressure = 85 mmHg.

^b^ Severe hypertension was defined as systolic BP ≥ 160 mmHg or diastolic BP ≥ 110 mmHg.

^c^ Proteinuria was defined as ≥ 300 mg/24 h; protein‐to‐creatinine ratio ≥ 30 mg/mmol; or ≥ ++ dipstick proteinuria.

^d^ Symptoms were one or more of: headache, visual disturbances, persistent right upper quadrant or epigastric pain, severe nausea or vomiting, chest pain, or dyspnoea.

^e^ Signs were in addition to severe hypertension, one or more of: eclampsia, placental abruption or pulmonary oedema.

^f^ Abnormal laboratory features were one or more of: elevated aspartate or alanine aminotransferase or lactate dehydrogenase (by local laboratory criteria) with symptoms, platelet count < 100000 × 109/L or serum creatinine > 2.26 mg/dL (> 200 μM).

^g^ Serious maternal complications included death, stroke, eclampsia, blindness, uncontrolled hypertension, the use of inotropic agents, pulmonary oedema, respiratory failure, myocardial ischaemia or infarction, hepatic dysfunction, hepatic haematoma or rupture, renal failure and transfusion.

^h^ Pregnancy loss defined as miscarriage, ectopic pregnancy, pregnancy termination, stillbirth or neonatal death.

^i^ High‐level neonatal care defined as greater‐than‐normal newborn care for more than 48 h, until 28 days of life or until discharge home, whichever was later.

^j^ Adjusted for maternal age, parity, body mass index (< 25 vs. ≥ 25 kg/m^2^), use of antihypertensive therapy at randomisation, gestational diabetes prior to randomisation, previous systolic BP ≥ 160 or diastolic BP ≥ 110 mmHg in this pregnancy, gestational age at recruitment, randomised group and centre (as random effects). Some outcomes were not assessed as there were few events.

That adverse perinatal events were more common among women with chronic hypertension who gave birth to SGA infants was unsurprising, but these women were also more likely to develop maternal manifestations of pre‐eclampsia, including severe maternal hypertension, a surrogate for stroke risk [5]. Data from this secondary analysis of the CHIPS trial support inclusion of FGR as a diagnostic criterion for pre‐eclampsia in women whose pregnancies are complicated by chronic hypertension to highlight the need for maternal surveillance [1].

## Author Contributions

P.v.D. and L.A.M. developed the question. T.L., with supervision by J.S., undertook the analyses. All authors contributed to writing the manuscript.

## Funding

This work was supported by Canadian Institutes of Health Research (grant number: MCT 87522).

## Conflicts of Interest

The authors declare no conflicts of interest.

## Data Availability

The data that support the findings of this study are available from the corresponding author upon reasonable request.
